# Supplementary material for: Immunogenicity of Endolysin PlyC
Source: Antibiotics (Basel). 2022 Jul 18;11(7):966. doi: 10.3390/antibiotics11070966 (PMC9312349; doi:10.3390/antibiotics11070966)
Supplement: Supplementary file 1 [file antibiotics-11-00966-s001.zip › antibiotics-1772484-supplementary.pdf]

Supplementary

Supplementary Table S1: Demonstration of PlyC antibacterial activity.

Bacterial host	Method(s)	Antibacterial activity	Reference
group A streptococci (*Streptococcus pyogenes)*	Plate assay; native PAGE electrophoresis on agarose embedded with sensitive bacteria	confirmed	Nelson et al., Proc Natl Acad Sci U S A. 2006
group A streptococci (*Streptococcus pyogenes)*	OD600 measurements in isotonic 30%sucrose/PBS w/v buffer	confirmed	Köller et al., Proteomics. 2008
group C streptococci (*Streptococcus equi, Streptococcus dysgalactiae*)	OD600 measurements in PBS	confirmed	Hoopes et al., Appl Environ Microbiol. 2009
Streptococcus: S. sobrinus, S. rattus, S. suis, S. pneumoniae, S. oralis, S. mutans, S. agalactiae, Staphylococcus: S. aureus, S. epidermidis, S. hyicus, Bacillus cereus, Enterococcus faecalis, Enterococcus faecium	OD600 measurements in PBS	not observed	Hoopes et al., Appl Environ Microbiol. 2009
group A streptococci (*Streptococcus pyogenes)*	OD600 measurements and plate count	confirmed	Chen et al., Sheng Wu Gong Cheng Xue Bao. 2009
group A streptococci (*Streptococcus pyogenes)*	OD600 measurements in PBS (with kinetics)	confirmed	McGowan et al., Proc Natl Acad Sci U S A. 2012
group A streptococci in biofilm (*Streptococcus pyogenes),*	microbiological cultures of bacterial cells recovered from biofilms	confirmed	Shen et al., J Antimicrob Chemother. 2013
group A streptococci, intracellular (*Streptococcus pyogenes),*	Co-culture with human epithelial cells and cell counting for recovered bacteria	confirmed	Shen et al., Elife. 2016

References for Supplementary Table S1

Nelson D, Schuch R, Chahales P, Zhu S, Fischetti VA. PlyC: a multimeric bacteriophage lysin. Proc Natl Acad Sci U S A. 2006;103(28):10765-70.

Köller T, Nelson D, Nakata M, Kreutzer M, Fischetti VA, Glocker MO, Podbielski A, Kreikemeyer B. PlyC, a novel bacteriophage lysin for compartment-dependent proteomics of group A streptococci. Proteomics. 2008;8(1):140-8.

Hoopes JT, Stark CJ, Kim HA, Sussman DJ, Donovan DM, Nelson DC. Use of a bacteriophage lysin, PlyC, as an enzyme disinfectant against Streptococcus equi. Appl Environ Microbiol. 2009;75(5):1388-94.

Chen W, Wang X, Wang P, Zhang D, Chen H, Ke W, Lu Y, Zhang J. [Expression, purification and characterization of bacteriophage lysin of Streptococcus in Escherichia coli]. Sheng Wu Gong Cheng Xue Bao. 2009;25(8):1267-72.

McGowan S, Buckle AM, Mitchell MS, Hoopes JT, Gallagher DT, Heselpoth RD, Shen Y, Reboul CF, Law RH, Fischetti VA, Whisstock JC, Nelson DC. X-ray crystal structure of the streptococcal specific phage lysin PlyC. Proc Natl Acad Sci U S A. 2012;109(31):12752-7.

Shen Y, Köller T, Kreikemeyer B, Nelson DC. Rapid degradation of Streptococcus pyogenes biofilms by PlyC, a bacteriophage-encoded endolysin. J Antimicrob Chemother. 2013;68(8):1818-24.

Shen Y, Barros M, Vennemann T, Gallagher DT, Yin Y, Linden SB, Heselpoth RD, Spencer DJ, Donovan DM, Moult J, Fischetti VA, Heinrich F, Lösche M, Nelson DC. A bacteriophage endolysin that eliminates intracellular streptococci. Elife. 2016;5:e13152.

Supplementary Table S2: Evaluation of anti-PlyC, anti-PlyCA, and anti-PlyCB IgG levels in human sera by normal distribution analysis. The Anderson-Darling analysis tests the null-hypothesis that a cumulative distribution curve does not vary from expected (i.e., Gaussian distribution). Similarly, the D'Agostina & Pearson analysis tests whether skewness and kurtosis of the data vary significantly from expected in Gaussian distribution.

	**PlyCB**	**PlyCA**	**PlyC**
**Anderson-Darling test**			
A2	3.391	0.5596	4.376
P value	<0.0001	0.1415	<0.0001
Passed normality test (alpha=0.05)?	No	Yes	No
**D'Agostino & Pearson test**			
K2	33.64	5.251	34.13
P value	<0.0001	0.0724	<0.0001
Passed normality test (alpha=0.05)?	No	Yes	No

Supplementary Table S3: Correlations of human serum IgG reactivity to PlyC and its domains: PlyC vs. PlyCA (first row), and PlyC vs. PlyCB (second row).

	**Spearman r**	**p-values**	**CI**	**Pearson r**	**p-values**	**CI**
PlyCA	0.24	0.078	-0.035 to 0.48	0.32	0.017	0.06 to 0.54
PlyCB	0.82	<0.0001	0.71 to 0.89	0.95	4.9E-30	0.92 to 0.97

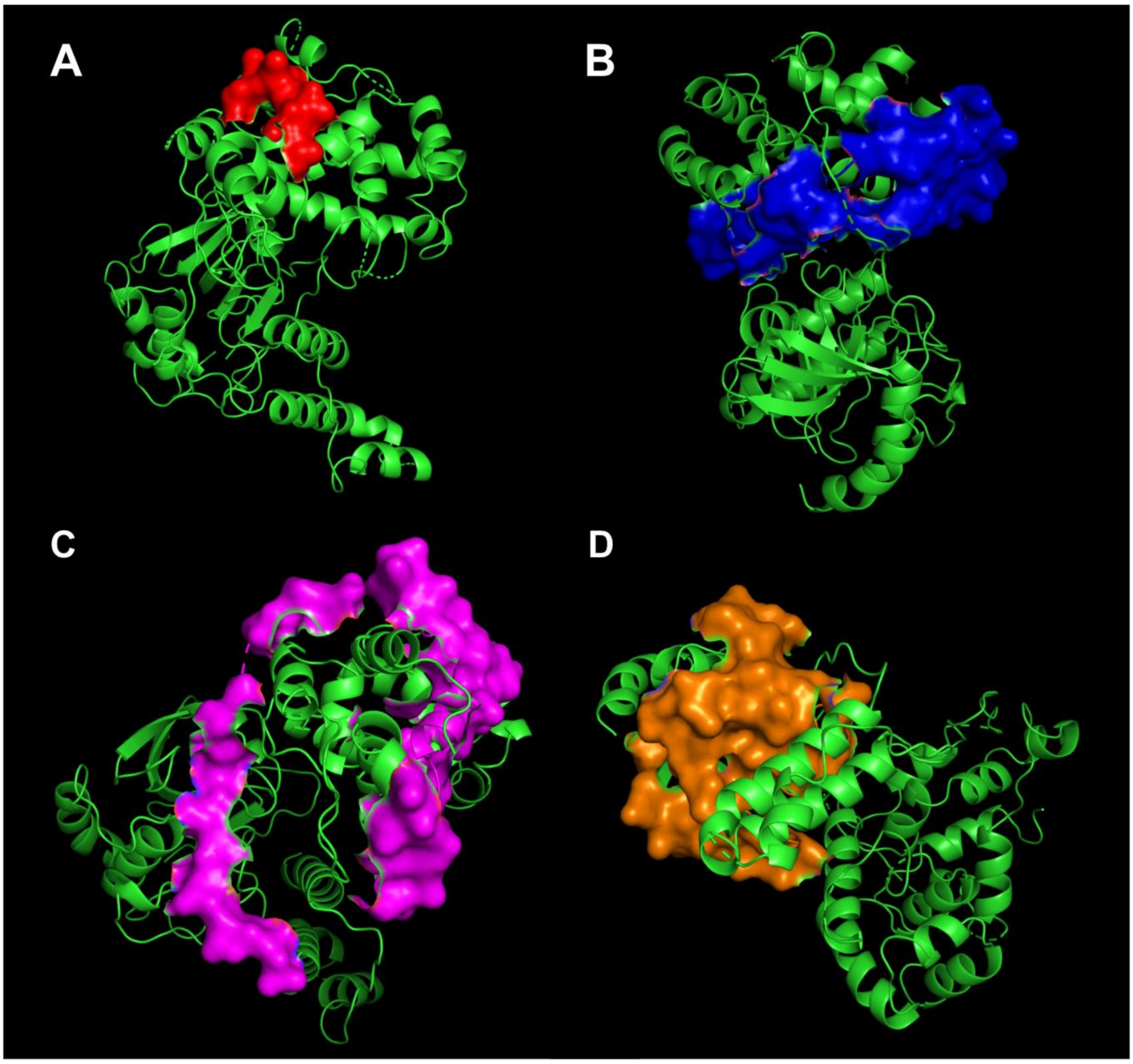

Supplementary Figure S1: Graphical representation of for immunogenic regions on PlyCA domain. Immunogenic regions are presented: 1- 9 aa (**A**), 91-146 aa (**B**), 171-226 aa (**C**), 351-406aa (**D**).

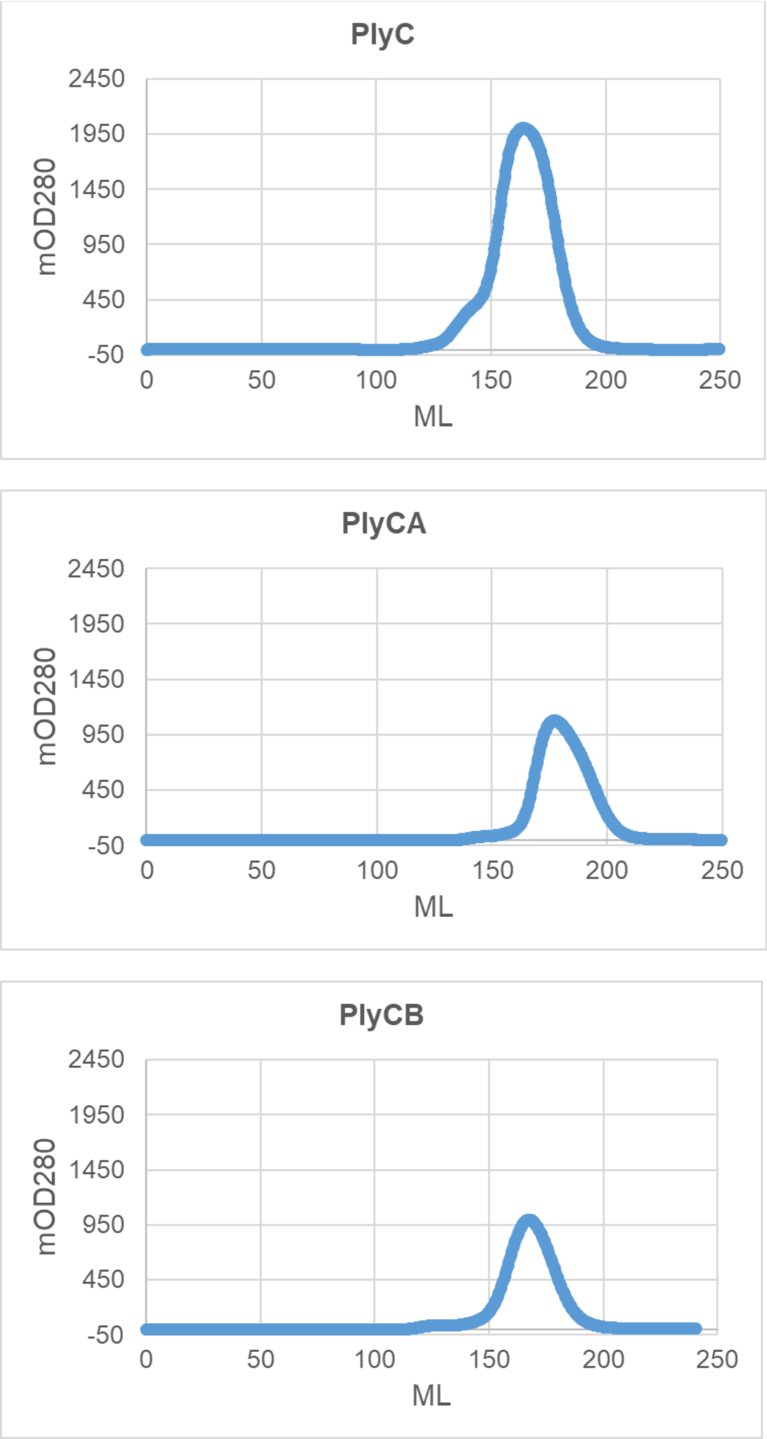

Supplementary Figure S2: Gel filtration of purified PlyC, PlyCA, and PlyCB. Purified proteins (10 ml each) were applied to 26/70 Sephacryl S-200 gel filtration column equilibrated in PBS. PlyC is a ~114 kDa holoenzyme composed of one PlyCA and eight PlyCB subunits. PlyCA is a ~50 kDa monomer. PlyCB is a ~64 kDa octamer, composed of eight identical PlyCB (8 kDa) monomers.
